# Supplementary material for: A focused multi-state model to estimate the pediatric and adolescent HIV epidemic in Thailand, 2005–2025
Source: PLoS One. 2022 Nov 17;17(11):e0276330. doi: 10.1371/journal.pone.0276330 (PMC9671429; doi:10.1371/journal.pone.0276330)
Supplement: S1 Appendix — (DOCX) [file pone.0276330.s001.docx]

**A focused multi-state model to estimate the pediatric and adolescent**

**HIV epidemic in Thailand, 2005 – 2025**

**Supporting information**

Sophie Desmonde

*et al.*

**TABLE OF CONTENTS**

**Methods**

**Table A –** Model input parameters

**Table B –** Number of children aged 0-15 years living with HIV in 2005 (results from adjacent tool used to derive model inputs)

**Table C –** Derivations of the overall mother-to-child-transmission rates

**Table D –** Summary of incidence inputs for scenario analyses on PrEP scale-up among MSM

**Table E –** Model-projected number of children and adolescents living with HIV according to care status

**Table F –** Summary of sensitivity analyses

**Figure A –** Model-projected versus PHIMS-reported mother-to-child-transmission rates, 2005-2015

**Figure B –** Model-projected versus reported proportion of CYHIV on ART among those who ever initiated treatment, 2008-2016

**Figure C –** Model-projected number of children and youth living with HIV by risk group, 2005-2025

**Figure D –** Model-projected age distribution among children and youth living with perinatally-acquire HIV, 2005-2025

**Figure E –** Number of new HIV infections among children and youth living with non-perinatally-acquired HIV, 2005-2025

**Figure F –** Number of children aged 0-14 years living with HIV in Thailand, 2005-2019 – comparison with Spectrum output

**Figure G –** Number of new infections among children aged 0-14 years in Thailand 2005-2019 – comparison with Spectrum output

**Figure H –** Number of children aged 10-19 years living with HIV in Thailand 2005-2019 – comparison with Spectrum output

**Figure I –** Number of children aged 15-24 years living with HIV in Thailand 2005-2019 – comparison with Spectrum output

**Figure J –** Number of new infections among adolescents aged 10-19 years in Thailand 2005-2019 – comparison with Spectrum output

**Figure K –** Number of new infections among adolescents aged 15-24 years in Thailand 2005-2019 – comparison with Spectrum output

**Figure L –** Sensitivity analysis: number of children aged 10-19 years living with HIV in Thailand 2005-2019 – comparison with Spectrum output.

**Figure M –** Sensitivity analysis: number of youth aged 15-24 years living with HIV in Thailand 2005-2019 – comparison with Spectrum output.

**Figure N.** ART coverage among CYHIV aged 0-25 years in Thailand, when increasing access to HIV testing among MSM and FSW, 2005-2025

**Figure O –** Number of people living with HIV among MSM aged 13-25 years in different PrEP uptake scenarios, 2015-2025

**INTRODUCTION**

This appendix is included to provide methodological details to supplement the description of the methods in the manuscript text, as well as additional model outputs and results.

**METHODS**

**Model structure**

The model structure is detailed in the manuscript, with an overview schematic in Manuscript Figure 1.

***Baseline cohort (2005)***

We began the running the model by estimating the number of CYHIV living in 2005. Because of incomplete assessment of HIV status among children early in the epidemic, for CYPHIV, we used UNAIDS-Spectrum estimates of new infant infections in each year from 1990 to 2005, to which we applied published survival rates, assuming the introduction of ART to prevent MTCT in 2001 (Supplemental Table B) ^[1, 2]^. In calibration analyses, we compared these projections to program data for each calendar year.

To model children and youth with non-perinatally acquired HIV (CYNPHIV) in 2005, we used demographic data from the Official Statistics Registration Systems and World Population Prospects ^[3, 4]^ to estimate the number of people of each age (13-24) in that year. We then sorted this overall population into risk groups (MSM, FSW, PWID and “other”) according to data available from the Bureau of Epidemiology, national surveys, and other published data. We next multiplied the number of people in each risk group who reported sexual activity to the national behavioral survey data by the reported HIV prevalence in each risk group ^[5-7]^. Detailed calculations are available in the methods section of the Supplemental Appendix.

***New infections (yearly)***

Children at risk for perinatally-acquired HIV face a modeled risk of acquiring HIV only during their first year of life, reflecting replacement feeding among most HIV-exposed infants, with short duration of breastfeeding if it occurs ^[8]^. In each calendar year, the number of new perinatal infections was modeled as a function of maternal HIV prevalence **among all women in Thailand, including foreign laborers**, prevention of MTCT (PMTCT) coverage, PMTCT regimen, and regimen-specific transmission rates (Supplemental Table C, Supplemental Figure A) ^[9]^. More specifically, between 2005-2010, the PMTCT regimen was Option A, comprised of AZT plus a single dose of NVP. From October 2010, the MOPH began rolling out Option B, ART for all HIV-infected pregnant women until cessation of breastfeeding, regardless of CD4 count. In 2013, this expanded to Option B+, involving lifelong ART for these women. MTCT rates under each of the three PMTCT regimens were derived from Spectrum ^[10]^.

Uninfected children, adolescents, and youth at risk for NPHIV (non-perinatally-acquired HIV) enter the model at age 13. We derived the number of youth at potential risk for HIV of each age, in each key population, for each calendar year using the same methodology as described above for the baseline cohort and then subtracting the number of CYHIV in each risk group who aged up in the model from the previous calendar year. For each age and calendar year, the number of new infections was calculated by multiplying the population at risk by the population-specific HIV incidence. HIV incidence among the remainder of the population (“other”) was estimated by subtracting the incidence in all other key populations from the UNAIDS incidence rate for 15-24-year-olds (available at https://aidsinfo.unaids.org). Incidence rates and detailed calculations are available in the methods section of Supplemental Appendix. Empiric data were available for years 2005-2020 for most parameters; for forward projections through 2025, we assumed minimal change from the most recent empiric data.

***Estimating the total number of CYHIV and new HIV infections in each year***

Total projected numbers of CYHIV in each calendar year depend on both new infections in the current year and survival from previous years; survival, in turn, varies by modeled health state. At the beginning of each cycle (year), surviving CYHIV from the previous calendar year “age up” to the next calendar year. For CYHIV who were on ART in the previous year, the model first assigns an age- and population-specific LTFU probability. For those who were in the “CYHIV LTFU” state, an age- and population-specific return to care probability is assigned. For each calendar year from 2005 to 2016, LTFU and return to care were derived from published data and then calibrated to yield numbers of CYHIV on ART that matched Thai National AIDS Program reports ^[11]^ (Supplemental Figure B).

Next, CYHIV with incident infections enter the model each year in the “CYHIV not on ART” health state, stratified by age and population. Among this cohort of children and youth with newly acquired HIV not on ART, the model then assigns age- and population-specific probabilities of having an HIV test and subsequently initiating ART, shifting CYHIV from the “not on ART’ to the “on ART” health state.

At the end of each cycle, the model assigns survival rates to each of the health states. Survival rates are stratified by mode of infection (perinatal vs non-perinatal), whether the cohort is on ART or not, and for CYPHIV, by age (<2 ; ≥2 years) for CYPHIV only. Those who survive then transition into the age + 1 year sub-state and a new cycle starts; those who were in the 25-year-old age sub-state exit the model by transitioning into the CYHIV “aged >25 years” absorbing state. At the end of each yearly cycle, the model tallies the total number of CYHIV; the number of children and youth deceased that year and cumulatively; and the number of new infections, by age, population, and care status (off ART, on ART or LTFU).

**Model input data**

Data used as input parameters for the model were reviewed by the Thai Working Group. Details of calculations are described below, with selected data inputs presented in Manuscript Table 1 and complete inputs in Supplemental Table A, below.

*Number of persons at risk each year*

Infants (<1 year of age) were at risk of acquiring HIV in their first year of life if they were born to women living with HIV. The number of infants at risk was obtained by multiplying the number of births each year by the reported HIV maternal prevalence for that year. For non-perinatal infections, the population was at risk if aged ≥ 13 years; this was calculated by multiplying the size of the population for each year by each of the proportions of 13-25-year-olds falling into the following sub groups:

- The proportion of the male population who were **men who have sex with men (MSM)** at risk of acquiring HIV was defined as the median proportion of males reporting sex with a male in the last 12 months between 2002-2016, as reported by the Bureau of Epidemiology for given age groups ^[5]^. Among this group, we next assigned the proportions designated as high- or low- risk MSM, with high-risk defined as having visited hotspots, as recommended by DOE. These proportions were calculated based on the following hypothesis: in Bangkok (8.5% of the male population), the high-risk:low-risk risk ratio was 1:1; in “tourist” provinces (5.5% of the male population), the ratio was 2:3, and in the rest of the country, the ratio was 3:7. We weighted these proportions by the population size to obtain national estimates by age group, available in Supplemental Table A.
- The proportion of the female population who were **female sex workers (FSW)** at risk of acquiring HIV was defined by age. For those <18 years, this was the median proportion of females in grade 8 reporting exchanging sex for money or gifts between 2002-2016, as reported by the Bureau of Epidemiology (BoE) (0.1%) ^[5]^. For those aged ≥18 years, we used the proportion previously derived by AEM (0.77%) ^[12]^.
- The proportion of the population who were **people who inject drugs (PWID)** at risk of acquiring HIV was derived based on data from the Drug Treatment Center, which reported 27.1% of PWID were <25 years in 2016 ^[6]^. We applied this proportion to the overall number of PWID in Thailand in 2016 to calculate the number of PWID <25 years which we then divided by the size of the overall population.
- Finally, for the remaining population that did not fall into any of the above, defined in the model as **“other,**” we assumed that the proportion at risk was 1 minus the sum of the above multiplied by the median proportion of those reporting to be sexually active 2002-2016, by age, according to the Integrated Biological and Behavioral Survey (IBBS) ^[7]^.

*HIV prevalence in 2005*

HIV prevalence in the first year of the model (2005) was obtained from different data sources depending on the risk group being simulated.

To assess the number of children with perinatally-acquired HIV, we first assumed that in the absence of ART, no person with CYHIV survived >15 years, meaning that the oldest person living with HIV was aged 15 years in 2005, thus born in 1990. We then used the Spectrum outputs of number of new HIV infections among 0-14-year-olds for each year between 1990 and 2004, and applied age-specific survival rates, accounting for ART introduction in 2001 ^[1]^. Details are available in Supplemental Table B. In this case, the model input was not a prevalence but the number of children surviving with perinatally acquired HIV in 2005.

For MSM, HIV prevalence in 2005 was derived from Silom Community Clinic in Bangkok data ^[13]^. Using data from the BoE describing the proportion of MSM living with HIV in different regions in Thailand, we applied a Bangkok versus the rest of the country ratio to the previously published data ^[5]^. Given that the data describes Bangkok hotspots, we considered the derived prevalence to be representative of high-risk MSM. We assumed the prevalence among low-risk MSM was 40% of that among high-risk MSM.

For FSW, we weighted the HIV prevalence estimate among 15-49 year olds (from the HIV Serological Surveillance Survey) by the HIV risk ratio among FSW aged 15-24 years compared to 25-49 years (from the Integrated Biological and Behavioral Survey (IBBS)) to obtain an estimate of 2.5% ^[5, 14]^ .

HIV prevalence data among young PWID in 2005 were difficult to identify, and so the Working Group recommended use of the 2014 IBBS estimate ^[7]^.

The HIV prevalence among the remainder of the population (“other” risk group) was derived based on Spectrum projections for ages 10-19 and 15-24 years; the size of key populations living with HIV, defined above, was subtracted from the projected number of children living with HIV and then divided by the size of the overall population ^[15]^.

*Proportion on ART in 2005*

The proportion of youth living with HIV already in care at the start of the simulation was calculated as the product of those who accessed an HIV diagnosis and those who subsequently initiated ART. The earliest available data for these indicators was 2008, which we applied to 2005. For ART use among those with perinatal infections, we used data from the Global AIDS Response Progress Reporting (GARPR). For ART use among those with non-perinatal infections, we divided the number on ART reported by the National AIDS Program (NAP) by the Spectrum estimate of those living with HIV.

*The mother-to-child transmission (MTCT) component*

Adjacent to the model, we built a specific MTCT input tool to derive a specific MTCT rate per year based on the coverage of prevention of MTCT (PMTCT) services. The Thai Perinatal HIV Intervention Monitoring System (PHIMS) provides an overall estimate of PMTCT coverage by year. Conditional upon receipt of a given regimen, we applied MTCT risks during pregnancy and delivery based on published clinical trials. These inputs are from the same data used in the Spectrum model, although do not fully incorporate changes made to Spectrum in 2019 accounting for MTCT risk for women with incident infection in late pregnancy or during breastfeeding ^[9]^. We assumed the introduction of Option B ART for all HIV-infected pregnant women until cessation of breastfeeding, regardless of CD4 count) in 2010, which was then replaced by Option B+, involving lifelong ART for these women, in 2013; we assumed that breastfeeding did not occur. Despite Thailand reaching the elimination of MTCT in 2016 (an MTCT risk <2%), we continued to simulate a small number of perinatally-acquired infections by carrying forward the 2015 maternal prevalence and estimated MTCT rate. These newly calculated estimates fit the adjusted national MTCT rates from the PHIMS report (Supplemental Table C and Supplemental Figure A).

*HIV incidence*

Among young MSM aged 13-21 years who were defined as high-risk, we used published HIV incidence inputs from two sources: the Silom Community Clinic voluntary counselling and testing services and the Bangkok MSM Cohort Study ^[16]^. Additional unpublished data from older MSM were also available from the Silom Community Clinic, allowing us to calculate a risk ratio of HIV incidence among MSM aged 15-21 years compared to 22-25 years, which we applied to the previous data ^[17]^. We then assumed that HIV incidence among low-risk MSM was one third of that estimated among high-risk MSM.

HIV incidence among FSW was from the IBBS; based on the Thai Working Group recommendations, we used venue-based incidence estimates ^[7]^.

Data on HIV incidence among PWID are limited. We used data from the Bangkok Tenofovir Study, a randomized placebo-controlled trial of PrEP for PWID conducted in 17 Bangkok Drug Treatment centers ^[6]^.

HIV incidence among the remainder of the population (the “other” risk group) was informed by the UNAIDS incidence rate for 15-24-year-olds, adjusted by subtracting the incidence among all other key populations. In sensitivity analyses, we conducted runs using the upper confidence interval bounds of the UNAIDS estimate.

*Accessing an HIV diagnosis*

The GARPR provided an estimate for HIV diagnosis access among youth living with perinatally-acquired HIV for years 2005-2015. Access to an HIV diagnosis was defined as the percentage of infants born to women living with HIV who receive an HIV virological test within 2 months of birth ^[11]^.

For youth in key populations (MSM, FSW and PWID), data on HIV testing were available from the IBBS. Among MSM, we used the percentage of MSM who received an HIV test in the last 12 months and knew their results (national mean among <25y); for FSW, we used the percentage of sex workers who received an HIV test in the last 12 months and who knew their results (national mean among <25y); and for PWID, we used the percentage of PWID who received an HIV test in the last 12 months and who knew their results ^[14]^. In sensitivity analyses, we used the UNAIDS indicator “HIV testing and status awareness for both the MSM and FSW population” in years 2019-2025.

For the remainder of the population, we used published data from a cohort of sexually active people aged 15-24 years in Chiang Mai ^[18]^. In projections for years after 2015, we multiplied the UNAIDS estimate for testing coverage by the percentage of young people who know about prevention.

*Linking to care: ART initiation*

For youth living with perinatally-acquired HIV, we calculated inputs for probability of initiating ART following a positive HIV test from the GARPR and UNAIDS estimates.

For youth living with non-perinatally-acquired HIV in modeled risk groups, we derived data on ART initiation from the National AIDS program (NAP) data ^[11]^. We note that data were not available for FSW prior to 2013, and the consensus among the pediatric Thai Working Group was to use 50%. Also, data on ART initiation for PWID were not available prior to 2014; we carried back the 2014 estimate to 2005.

For the remainder of the population (“other” risk group), we derived inputs based on UNAIDS estimates and the NAP data. UNAIDS provides an overall estimate of those on ART among those living with HIV aged 15-49 years (https://aidsinfo.unaids.org/). The NAP reports 70% of those aged 15-49 years access HIV testing ^[11]^. We calculated the probability of initiating ART after being tested by dividing the percentage on ART by the percentage accessing an HIV test.

*Loss to follow-up and return to care*

Loss to follow-up (LTFU) rate was derived by age group and was assumed to be 4-10% in the base case, the same for each of the six modeled risk groups. Based on the NAP data reporting the number of patients who stopped ART by year, we calculated the LTFU rate as the sum of this number and those already LTFU divided by the total number of participants in the program ^[11]^. Return to care was calibrated to match the NAP yearly estimates : to perform this, for each year we ran the model with no return to care, and calculated the proportion among those LTFU who must have returned to care for ART coverage to match the NAP estimate for the given year (Supplemental Figure B).

**REFERENCES**

1. Becquet R, Marston M, Dabis F, Moulton LH, Gray G, Coovadia HM, et al. **Children who acquire HIV infection perinatally are at higher risk of early death than those acquiring infection through breastmilk: a meta-analysis**. *PloS One* 2012; 7(2):e28510.

2. Mahy M, Stover J, Kiragu K, Hayashi C, Akwara P, Luo C, et al. **What will it take to achieve virtual elimination of mother-to-child transmission of HIV? An assessment of current progress and future needs**. *Sex Transm Infect* 2010; 86 Suppl 2:ii48-55.

3. Official Statistics Thailand. **Official Statistics Registration Systems** In; 2017.

4. United Nations, Department of Economic and Social Affairs, Population Division. **World Population Prospects: The 2017 Revision, custom data acquired via website**. In; 2017.

5. Bureau of Epidemiology. **HIV Surveillance System**. In; 2010-2014.

6. Choopanya K, Martin M, Suntharasamai P, Sangkum U, Mock PA, Leethochawalit M, et al. **Antiretroviral prophylaxis for HIV infection in injecting drug users in Bangkok, Thailand (the Bangkok Tenofovir Study): a randomised, double-blind, placebo-controlled phase 3 trial**. *Lancet* 2013; 381(9883):2083-2090.

7. Bureau of Epidemiology, Thailand MoPH and Department of Health. **Integrated Bio-Behavioral Survey**. In; 2016.

8. Plipat T, Naiwatanakul T, Rattanasuporn N, Sangwanloy O, Amornwichet P, Teeraratkul A, et al. **Reduction in mother-to-child transmission of HIV in Thailand, 2001-2003: Results from population-based surveillance in six provinces**. *AIDS* 2007; 21(2):145-151.

9. Mahy M, Penazzato M, Ciaranello A, Mofenson L, Yianoutsos CT, Davies MA, et al. **Improving estimates of children living with HIV from the Spectrum AIDS Impact Model**. *AIDS* 2017; 31 Suppl 1:S13-S22.

10. Stover J, Johnson P, Zaba B, Zwahlen M, Dabis F, Ekpini RE. **The Spectrum projection package: improvements in estimating mortality, ART needs, PMTCT impact and uncertainty bounds**. *Sex Transm Infect* 2008; 84 Suppl 1:i24-i30.

11. National AIDS Program. In; 2017.

12. Brown T, Peerapatanapokin W. **The Asian Epidemic Model: a process model for exploring HIV policy and programme alternatives in Asia**. *Sex Transm Infect* 2004; 80 Suppl 1:i19-24.

13. van Griensven F, Varangrat A, Wimonsate W, Tanpradech S, Kladsawad K, Chemnasiri T, et al. **Trends in HIV Prevalence, Estimated HIV Incidence, and Risk Behavior Among Men Who Have Sex With Men in Bangkok, Thailand, 2003-2007**. *J Acquir Immune Defic Syndr* 2010; 53(2):234-239.

14. Integrated Bio-Behavioral Survey. 2010-2014.

15. World Health Organisation. **UNAIDS Data 2017**. In; 2017.

16. van Griensven F, Holtz TH, Thienkrua W, Chonwattana W, Wimonsate W, Chaikummao S, et al. **Temporal trends in HIV-1 incidence and risk behaviours in men who have sex with men in Bangkok, Thailand, 2006-13: an observational study**. *Lancet HIV* 2015; 2(2):e64-70.

17. van Griensven F, Mock PA, Benjarattanaporn P, Premsri N, Thienkrua W, Sabin K, et al. **Estimating recent HIV incidence among young men who have sex with men: Reinvigorating, validating and implementing Osmond's algorithm for behavioral imputation**. *PLoS One* 2018; 13(10):e0204793.

18. Musumari PM, Tangmunkongvorakul A, Srithanaviboonchai K, Yungyuankul S, Techasrivichien T, Suguimoto SP, et al. **Prevalence and Correlates of HIV Testing among Young People Enrolled in Non-Formal Education Centers in Urban Chiang Mai, Thailand: A Cross-Sectional Study**. *PloS one* 2016; 11(4):e0153452.
