# Supplementary material for: A focused multi-state model to estimate the pediatric and adolescent HIV epidemic in Thailand, 2005–2025
Source: PLoS One. 2022 Nov 17;17(11):e0276330. doi: 10.1371/journal.pone.0276330 (PMC9671429; doi:10.1371/journal.pone.0276330)
Supplement: S1 Table — (DOCX) [file pone.0276330.s002.docx]

**Table A. Model input parameters (including those shown in Manuscript Table 1**

| **Parameter** | **Value** | | | | | | **Source** | |
| --- | --- | --- | --- | --- | --- | --- | --- | --- |
| **I - Demographic distribution of sub-population data (remainder of 100% are not in any category and not sexually active)** | | | | | | | | |
|  | | *13-14y* | | *15-17y* | *18-19y* | *≥20y* | |  |
| High-risk MSM | | 0.1% | | 0.3% | 0.3% | 0.7% | | Derived from BoE. HIV Surveillance System. 2010-2014[1] |
| Low-risk MSM | | 0.1% | | 0.7% | 0.7% | 1.4% | |  |
| FSW | | 0.1% | | 0.1% | 0.4% | 0.4% | |  |
| PWID | | 0.1% | | 0.1% | 0.1% | 0.1% | | Choopanya K, 2013 [2] |
| Other youth | | 3.1% | | 30.6% | 30.6% | 84.2% | | Remainder, weighted by sexual activity, BoE, IBBS 2016 [3] |
| **II - HIV prevalence among those with non-perinatally-acquired HIV in 2005** | | | | | | | | |
| CYPHIV | | See Supplemental Table B | | | | | |  |
| High-risk MSM | | 4.8-8.0 | | | | | | Derived from van Griensven F, 2010 [4] |
| Low-risk MSM | | 3.2-19.2 | | | | | | Derived from van Griensven F, 2010 [4] |
| FSW | | 2.5 | | | | | | BoE. HIV Surveillance System. 2010-2014, IBBS 2013 [1, 5] |
| PWID | | 9.2 | | | | | | BoE, IBBS 2016 [3] |
| Other youth | | 0.1-0.5 | | | | | | Derived from UNAIDS Data 2017 [6] |
| **III – New MTCT HIV infections (See Supplemental Table C for detailed calculations)** | | | | | | | | |
| Maternal prevalence | | | 0.9-6.0% (range by year) | | | | |  |
| PMTCT coverage | | Overall: 89.6 - 95.6% (range by year)  Option A: 86.9-94.7%   Option B (2010-2013 only): 84.0-87.0% | | | | | | Derived from PHIMS report, [1] and Mahy M, 2017 [7] |
| MTCT rates | | Option A: 4.1%  Option B: 1.9%  Option B+: 0.7%  No PMTCT: 22.0% | | | | | | Mahy M, 2017 [7] |
| **IV - Incidence rates among those with non-perinatally-acquired HIV (%, yearly)** | | | | | | | | |
| High-risk MSM | | 1.8-4.0 | | | | | | Derived from van Griensven F, 2015 [8] and van Griensven F, 2018 [9] |
| Low-risk MSM | | 0.7-1.3 | | | | | | Derived from van Griensven F, 2015[8] and van Griensven F, 2018 [9] |
| FSW | | 0.6 | | | | | | BoE, IBBS 2016 [3] |
| PWID | | 0.7 | | | | | | Choopanya K, 2013 [2] |
| Other youth | | 0.008-0.08 | | | | | | Derived from UNAIDS-Spectrum estimates |
| **V - Access to HIV testing (%, yearly)** | | | | | | | | |
| CYPHIV | | 54-94 | | | | | | GARPR indicators |
| High-risk MSM | | 13-29 | | | | | | National AIDS Program 2017 [10], P. Chaiphosri, 2016 [10, 11] |
| Low-risk MSM | |  |  |  |  |  |  |  |
| FSW | | 40-60 | | | | | | National AIDS Program 2017 [10] |
| PWID | | 25 | | | | | | BoE, IBBS 2016 [3] |
| Other youth | | 28-40 | | | | | | Musumari PM, 2016 [12] |

**Table A. Continued**

| **Parameter** | **Value** | | **Source** | |
| --- | --- | --- | --- | --- |
| **VI - Access to ART among those tested (yearly)** | | | | |
| CYPHIV | 52-85 | | National AIDS Program 2017 [10] | |
| High-risk MSM | 35-66 | | National AIDS Program 2017 [10], P. Chaiphosri, 2016 [11] | |
| Low-risk MSM |  | |  | |
| FSW | 50-67 | | National AIDS Program 2017 [10] | |
| PWID | 44 | | National AIDS Program 2017 [10] | |
| Other youth | 18-84 | | Derived from UNAIDS-Spectrum estimates | |
|  |  | |  | |
| **VI – Survival rates (yearly)** | | | | |
| CYPHIV < 2 years | |  | |  |
| On ART | | 0.96 | | Violari A, 2008[13] |
| Off ART | | 0.69 | |  |
| CYPHIV ≥ 2 years | |  | |  |
| On ART | | 0.99 | |  |
| Off ART | | 0.92 | |  |
| CYNPHIV | |  | |  |
| On ART | | 0.96 | | Teeraananchai S, 2017[14] |
| Off ART | | 0.94 | | Teeraananchai S, 2017[14] |

**MSM:** men who have sex with men, **FSW:** female sex workers, **PWID:** people who inject drugs, **CYPHIV:** children and youth living with perinatally acquired HIV, **MTCT:** mother-to-child transmission, **PMTCT:** prevention of mother-to-child transmission, **ART:** antiretroviral therapy.

1. Bureau of Epidemiology. HIV Surveillance System. **2010-2014**.

2. Choopanya K, Martin M, Suntharasamai P, et al. Antiretroviral prophylaxis for HIV infection in injecting drug users in Bangkok, Thailand (the Bangkok Tenofovir Study): a randomised, double-blind, placebo-controlled phase 3 trial. Lancet **2013**; 381(9883): 2083-90.

3. Bureau of Epidemiology, Thailand MoPH and Department of Health. Integrated Bio-Behavioral Survey. **2016**.

4. van Griensven F, Varangrat A, Wimonsate W, et al. Trends in HIV Prevalence, Estimated HIV Incidence, and Risk Behavior Among Men Who Have Sex With Men in Bangkok, Thailand, 2003-2007. J Acquir Immune Defic Syndr **2010**; 53(2): 234-9.

5. Integrated Bio-Behavioral Survey. **2010-2014**.

6. World Health Organisation. UNAIDS Data 2017. Available at: <http://www.unaids.org/sites/default/files/media_asset/2017_data-book_en.pdf>.

7. Mahy M, Penazzato M, Ciaranello A, et al. Improving estimates of children living with HIV from the Spectrum AIDS Impact Model. AIDS **2017**; 31 Suppl 1: S13-S22.

8. van Griensven F, Holtz TH, Thienkrua W, et al. Temporal trends in HIV-1 incidence and risk behaviours in men who have sex with men in Bangkok, Thailand, 2006-13: an observational study. Lancet HIV **2015**; 2(2): e64-70.

9. van Griensven F, Mock PA, Benjarattanaporn P, et al. Estimating recent HIV incidence among young men who have sex with men: Reinvigorating, validating and implementing Osmond's algorithm for behavioral imputation. PLoS One **2018**; 13(10): e0204793.

10. National AIDS Program. **2017**.

11. P. Chaiphosri, S. Jantaramanee, Tanpradech S, et al. Utilization of multiple strategic information to inform program barriers towards the "Ending AIDS by 2020" in Bangkok, Thailand AIDS 2016. Durban, South Africa, **2016**.

12. Musumari PM, Tangmunkongvorakul A, Srithanaviboonchai K, et al. Prevalence and Correlates of HIV Testing among Young People Enrolled in Non-Formal Education Centers in Urban Chiang Mai, Thailand: A Cross-Sectional Study. PloS one **2016**; 11(4): e0153452.

13. Violari A, Cotton MF, Gibb DM, et al. Early antiretroviral therapy and mortality among HIV-infected infants. N Engl J Med **2008**; 359(21): 2233-44.

14. Teeraananchai S, Chaivooth S, Kerr SJ, et al. Life expectancy after initiation of combination antiretroviral therapy in Thailand. Antivir Ther **2017**; 22(5): 393-402.
