# Supplementary material for: A focused multi-state model to estimate the pediatric and adolescent HIV epidemic in Thailand, 2005–2025
Source: PLoS One. 2022 Nov 17;17(11):e0276330. doi: 10.1371/journal.pone.0276330 (PMC9671429; doi:10.1371/journal.pone.0276330)
Supplement: S2 Table — (DOCX) [file pone.0276330.s003.docx]

**Table B. Number of children aged 0-15 years living with HIV in 2005 (results from adjacent tool used to derive model inputs)**

| **Yearly survival rate off ART** | | | | | <2 years: 0.69  ≥ 2 years: 0.92 | |
| --- | --- | --- | --- | --- | --- | --- |
| **Yearly survival rate on ART (since 2001)** | | | | | <2 years: 0.96[1]  ≥ 2 years: 0.99[2] | |
| **Age in 2005** | **Year of birth** | | **Number of new HIV infections in year of birth estimated by Spectrum** | | | **Number of children living with HIV in 2005** |
| 1 | 2004 | | 2,300 | | | 1,759 |
| 2 | 2003 | | 2,500 | | | 1,780 |
| 3 | 2002 | | 3,000 | | | 698 |
| 4 | 2001 | | 3,400 | | | 743 |
| 5 | 2000 | | 3,700 | | | 759 |
| 6 | 1999 | | 4,300 | | | 2,151 |
| 7 | 1998 | | 4,400 | | | 2,043 |
| 8 | 1997 | | 4,400 | | | 1,880 |
| 9 | 1996 | | 4,300 | | | 1,690 |
| 10 | 1995 | | 4,100 | | | 1,483 |
| 11 | 1994 | | 3,800 | | | 1,264 |
| 12 | 1993 | | 3,300 | | | 1,010 |
| 13 | 1992 | | 2,600 | | | 732 |
| 14 | 1991 | | 1,700 | | | 440 |
| 15 | 1990 | | 1,000 | | | 238 |
| **Total** | |  | |  | | 18,671 |

**ART:** antiretroviral therapy.

1. Violari A, Cotton MF, Gibb DM, Babiker AG, Steyn J, Madhi SA, et al. Early antiretroviral therapy and mortality among HIV-infected infants. N Engl J Med. 2008;359(21):2233-44.

2. Teeraananchai S, Chaivooth S, Kerr SJ, Bhakeecheep S, Avihingsanon A, Teeraratkul A, et al. Life expectancy after initiation of combination antiretroviral therapy in Thailand. Antivir Ther. 2017;22(5):393-402. doi: 10.3851/IMP3121. PubMed PMID: 28054931.
