# Supplementary material for: A focused multi-state model to estimate the pediatric and adolescent HIV epidemic in Thailand, 2005–2025
Source: PLoS One. 2022 Nov 17;17(11):e0276330. doi: 10.1371/journal.pone.0276330 (PMC9671429; doi:10.1371/journal.pone.0276330)
Supplement: S3 Table — (DOCX) [file pone.0276330.s004.docx]

**Table C – Derivations of the overall mother-to-child-transmission rates**

|  | **2005** | **2006** | | **2007** | **2008** | **2009** | **2010** | **2011** | **2012** | **2013** | **2014** | **2015** |
| --- | --- | --- | --- | --- | --- | --- | --- | --- | --- | --- | --- | --- |
| **PMTCT coverage** | | | | | | | | | | | | |
| Option A | 89.6% | | 91.6% | 91.6% | 93.6% | 91.6% | 6.6%* | 6.2%* | 5.8%* | 5.5% | 5.1% | 4.7% |
| Option B^†^ |  | |  |  |  |  | 84.0% | 88.0% | 87.0% | 0.0% | 0.0% | 0.0% |
| Option B+ |  | |  |  |  |  |  |  |  | 89.0% | 90.0% | 91.0% |
| Total coverage | 89.6% | | 91.6% | 91.6% | 93.6% | 91.6% | 91.0% | 94.0% | 93.0% | 94.4% | 95.3% | 95.6% |
| **MTCT rates** | | | | | | | | | | | | |
| No PMTCT | 22.0% | | | | | | | | | | | |
| Option A | 4.1% | | | | | | | | | | | |
| Option B | 1.9% | | | | | | | | | | | |
| Option B+ | 0.7% | | | | | | | | | | | |
| Modeled MTCT rate | 6.0% | | 5.6% | 5.6% | 5.3% | 5.6% | 3.9% | 3.3% | 3.5% | 2.2% | 2.0% | 2.0% |
| National MTCT rate | 4.5% | | 3.5% | 5.3% | 4.6% | 3.9% | 3.4% | 3.1% | 2.7% | 2.3% | 2.0% | 2.0% |

**PMTCT:** prevention of mother-to-child transmission, **MTCT:** mother-to-child transmission.

* Estimates for Option A coverage in 2010-2012 was calculated based on mean trend in coverage reduction applied backwards since 2013

† Estimates for Option B coverage were unavailable in the literature. We calculated these as the reported overall PMTCT coverage – Option A coverage.

All values are rounded to the nearest tenth of a percent
