# Supplementary material for: A focused multi-state model to estimate the pediatric and adolescent HIV epidemic in Thailand, 2005–2025
Source: PLoS One. 2022 Nov 17;17(11):e0276330. doi: 10.1371/journal.pone.0276330 (PMC9671429; doi:10.1371/journal.pone.0276330)
Supplement: S4 Table — (DOCX) [file pone.0276330.s005.docx]

|  | **PrEP acceptance (%, source)** | **Incidence reduction among PrEP users [1]** | **Weighted incidence among MSM  (PrEP users and non-users)** | | | |
| --- | --- | --- | --- | --- | --- | --- |
|  |  |  | **High-risk (13-21 yo)** | **High-risk (22-25 yo)** | **Low-risk (13-21 yo)** | **Low-risk (22-25 yo)** |
| No Prep |  |  | 1.64% | 1.02% | 0.82% | 0.52% |
| Prep – sc1 | 35% [2] | 49% | 1.35% | 0.84% | 0.45% | 0.28% |
| Prep – sc2 | 41% [3] | 49% | 1.30% | 0.81% | 0.43% | 0.27% |
| Prep – sc3 | 75% [4] | 49% | 1.01% | 0.63% | 0.34% | 0.21% |
| Prep – sc4 | 100% | 49% | 0.80% | 0.50% | 0.27% | 0.17% |

**Table D – Summary of incidence inputs for scenario analyses on PrEP scale-up among MSM**

**PrEP:** pre-exposure prophylaxis, **MSM:** men who have sex with men.

1. Choopanya K, Martin M, Suntharasamai P, Sangkum U, Mock PA, Leethochawalit M, et al. Antiretroviral prophylaxis for HIV infection in injecting drug users in Bangkok, Thailand (the Bangkok Tenofovir Study): a randomised, double-blind, placebo-controlled phase 3 trial. Lancet. 2013;381(9883):2083-90. doi: 10.1016/S0140-6736(13)61127-7. PubMed PMID: 23769234.

2. Sineath RC, Finneran C, Sullivan P, Sanchez T, Smith DK, Griensven F, et al. Knowledge of and interest in using preexposure prophylaxis for HIV prevention among men who have sex with men in Thailand. J Int Assoc Provid AIDS Care. 2013;12(4):227-31. doi: 10.1177/2325957413488184. PubMed PMID: 23708677.

3. Yang D, Chariyalertsak C, Wongthanee A, Kawichai S, Yotruean K, Saokhieo P, et al. Acceptability of pre-exposure prophylaxis among men who have sex with men and transgender women in Northern Thailand. PloS one. 2013;8(10):e76650. doi: 10.1371/journal.pone.0076650. PubMed PMID: 24116132; PubMed Central PMCID: PMCPMC3792988.

4. Grant RM, Anderson PL, McMahan V, Liu A, Amico KR, Mehrotra M, et al. Uptake of pre-exposure prophylaxis, sexual practices, and HIV incidence in men and transgender women who have sex with men: a cohort study. Lancet Infect Dis. 2014;14(9):820-9. doi: 10.1016/S1473-3099(14)70847-3. PubMed PMID: 25065857; PubMed Central PMCID: PMCPMC6107918.
