# Supplementary material for: A focused multi-state model to estimate the pediatric and adolescent HIV epidemic in Thailand, 2005–2025
Source: PLoS One. 2022 Nov 17;17(11):e0276330. doi: 10.1371/journal.pone.0276330 (PMC9671429; doi:10.1371/journal.pone.0276330)
Supplement: S5 Table — (DOCX) [file pone.0276330.s006.docx]

**Table E – Model-projected number of children and adolescents living with HIV according to care status**

|  | **2005** | **2010** | **2015** | **2020** | **2025** |
| --- | --- | --- | --- | --- | --- |
| **On ART** | 13,959 | 23,096 | 23,689 | 18,845 | 12,892 |
| CYPHIV | 8,661 | 14,309 | 13,253 | 9,947 | 5,612 |
| MSM | 965 | 2,216 | 4,114 | 6,307 | 5,965 |
| *High-risk MSM* | 530 | 1,261 | 2,187 | 3,615 | 3,421 |
| *Low-risk MSM* | 435 | 955 | 1,927 | 2,692 | 2,544 |
| FSW | 240 | 531 | 537 | 526 | 495 |
| PWID | 259 | 464 | 318 | 186 | 182 |
| Other youth | 3,833 | 5,576 | 5,080 | 1,868 | 638 |
| **Returned to care** | 2,015 | 716 | 1,415 | 1,544 | 982 |
| CYPHIV | 650 | 518 | 1,019 | 1,006 | 562 |
| MSM | 232 | 48 | 158 | 346 | 342 |
| *High-risk MSM* | 126 | 27 | 91 | 199 | 197 |
| *Low-risk MSM* | 106 | 21 | 67 | 147 | 145 |
| FSW | 28 | 13 | 20 | 29 | 27 |
| PWID | 51 | 14 | 19 | 12 | 12 |
| Other youth | 1,054 | 123 | 199 | 151 | 39 |
| **Stopped ART** | 628 | 1,577 | 2,015 | 2,186 | 1,430 |
| CYPHIV | 304 | 1,068 | 1,683 | 1,319 | 740 |
| MSM | 55 | 124 | 350 | 585 | 564 |
| *High-risk MSM* | 30 | 70 | 200 | 336 | 324 |
| *Low-risk MSM* | 25 | 54 | 150 | 249 | 240 |
| FSW | 7 | 32 | 44 | 49 | 46 |
| PWID | 12 | 31 | 34 | 20 | 18 |
| Other youth | 250 | 322 | 446 | 214 | 62 |
| **Never initiated ART** | 50,340 | 26,686 | 11,860 | 8,180 | 7,334 |
| CYPHIV | 9,466 | 1,145 | 31 | 17 | 16 |
| MSM | 8,232 | 11,237 | 10,527 | 7,124 | 6,475 |
| *High-risk MSM* | 4,566 | 6,418 | 6,331 | 4,066 | 3,694 |
| *Low-risk MSM* | 3,666 | 4,819 | 4,196 | 3,058 | 2,781 |
| FSW | 726 | 513 | 276 | 218 | 203 |
| PWID | 1,235 | 588 | 399 | 345 | 327 |
| Other youth | 30,681 | 13,203 | 3,850 | 476 | 313 |

**ART:** antiretroviral therapy, **CYPHIV:** children and youth living with perinatally acquired HIV, **MSM:** men who have sex with men, **FSW:** female sex workers, **PWID:** people who inject drugs.
