# Supplementary material for: A focused multi-state model to estimate the pediatric and adolescent HIV epidemic in Thailand, 2005–2025
Source: PLoS One. 2022 Nov 17;17(11):e0276330. doi: 10.1371/journal.pone.0276330 (PMC9671429; doi:10.1371/journal.pone.0276330)
Supplement: S6 Table — (DOCX) [file pone.0276330.s007.docx]

**Table F – Summary of sensitivity analyses**

|  | **2005** | **2010** | **2015** | **2020** | | **2025** | |
| --- | --- | --- | --- | --- | --- | --- | --- |
| **Overall number of children and adolescents living with HIV** | | | |  | |  | |
| Basecase | 66942 | 52074 | 41976 | 30755 | | 22638 | |
| High:low MSM 1 | 68727 | 54826 | 44957 | 33669 | | 25300 | |
| High:low MSM 2 | 67862 | 53493 | 43512 | 32256 | | 23991 | |
| 2005 prevalence x 2 | 103990 | 58890 | 42441 | 30755 | | 22638 | |
| HIV test MSM | 66942 | 52074 | 41976 | 30764 | | 22692 | |
| **Overall number of adolescents aged 10-19 years living with HIV** | | | | |  |  | |
| Basecase | 13589 | 17862 | 15300 | 7776 | | 4393 | |
| High:low MSM 1 | 14033 | 18569 | 15943 | 8374 | | 4948 | |
| High:low MSM 2 | 13818 | 18226 | 15632 | 8083 | | 4673 | |
| 2005 prevalence x 2 | 17686 | 18173 | 15300 | 7776 | | 4393 | |
| HIV test MSM | 13589 | 17862 | 15300 | 7778 | | 4400 | |
| **Overall number of adolescents and youth aged 15-24 years living with HIV** | | | | | | |  |
| Basecase | 40577 | 31006 | 31301 | 24666 | | 17370 | |
| High:low MSM 1 | 42087 | 33327 | 33788 | 27079 | | 19569 | |
| High:low MSM 2 | 41355 | 32202 | 32583 | 25909 | | 18484 | |
| 2005 prevalence x 2 | 71302 | 34023 | 31536 | 24666 | | 17370 | |
| HIV test MSM | 40577 | 31006 | 31301 | 24674 | | 17412 | |

**High:low MSM 1**: ratio of high-risk to low-risk MSM assumed to be 1:1 throughout Thailand. **High:low MSM 2**: ratio assumed to be 1:1 in Bangkok and tourist provinces, and 2:3 elsewhere. **2005 prevalence x 2**: prevalence of HIV among children and youth aged 0-25 in 2005 (start of model simulation) is doubled**. HIV test MSM**: Assume 42.9% of MSM are aware of HIV status based on UNAIDS indicator data from 2019 onward.
