# Supplementary figures and images for: A focused multi-state model to estimate the pediatric and adolescent HIV epidemic in Thailand, 2005–2025
Source: PLoS One. 2022 Nov 17;17(11):e0276330. doi: 10.1371/journal.pone.0276330 (PMC9671429; doi:10.1371/journal.pone.0276330)

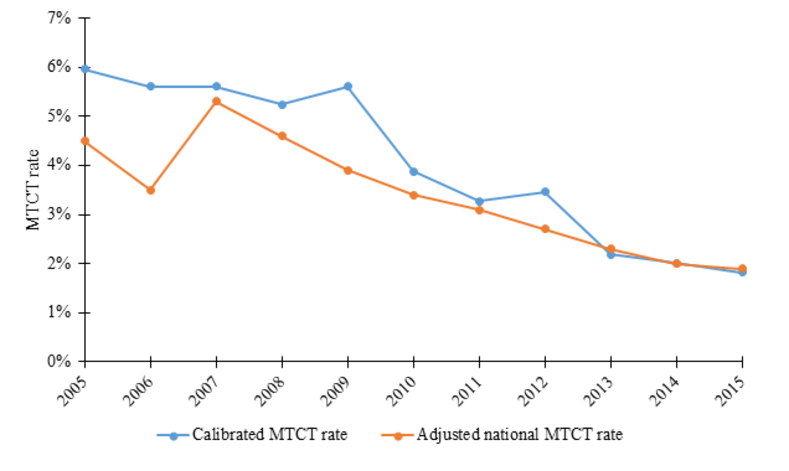

Supplement: S1 Fig — (TIF) [file pone.0276330.s008.tif]

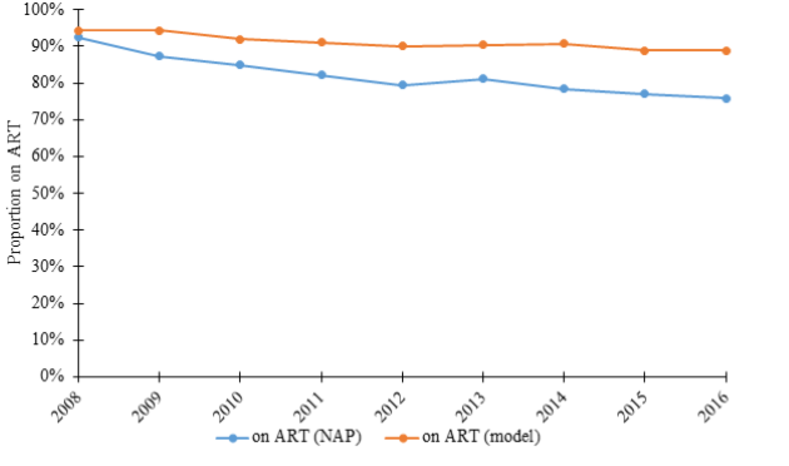

Supplement: S2 Fig — (TIF) [file pone.0276330.s009.tif]

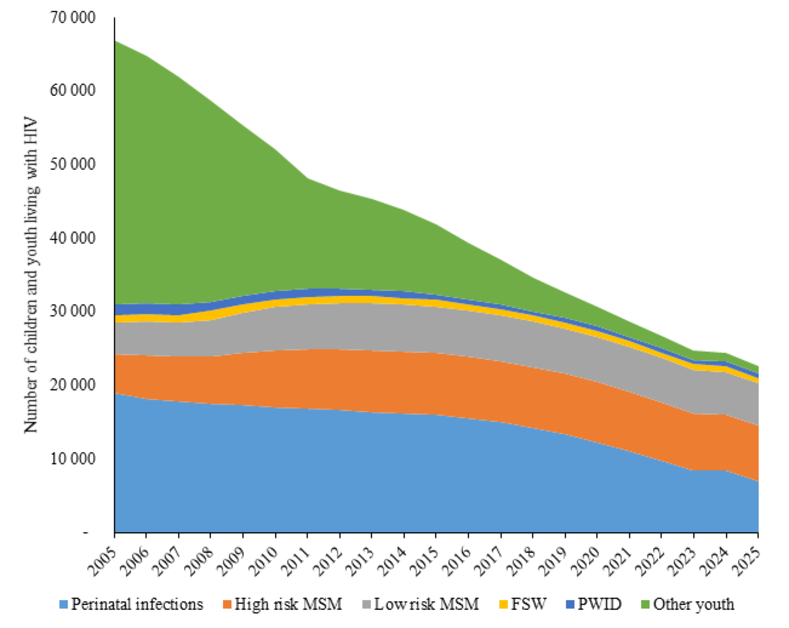

Supplement: S3 Fig — (TIF) [file pone.0276330.s010.tif]

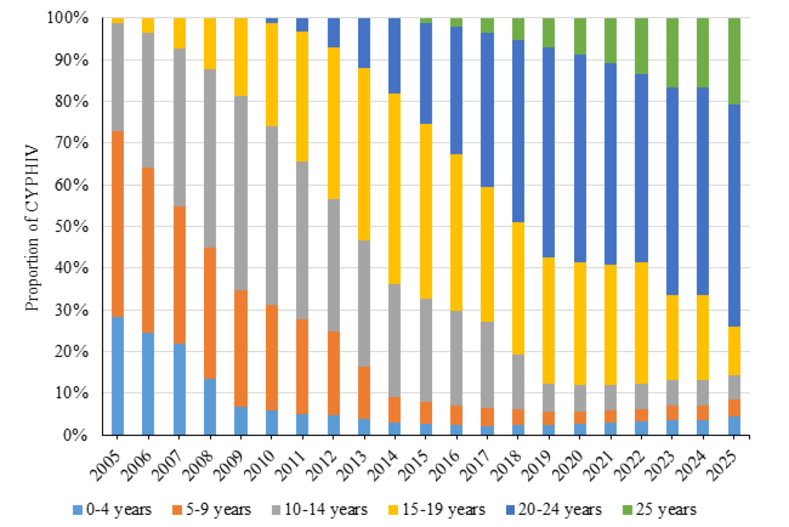

Supplement: S4 Fig — (TIF) [file pone.0276330.s011.tif]

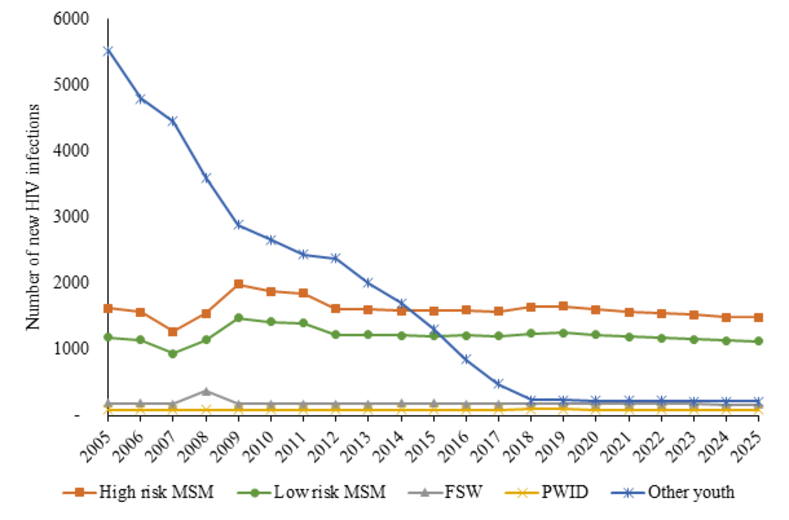

Supplement: S5 Fig — (TIF) [file pone.0276330.s012.tif]

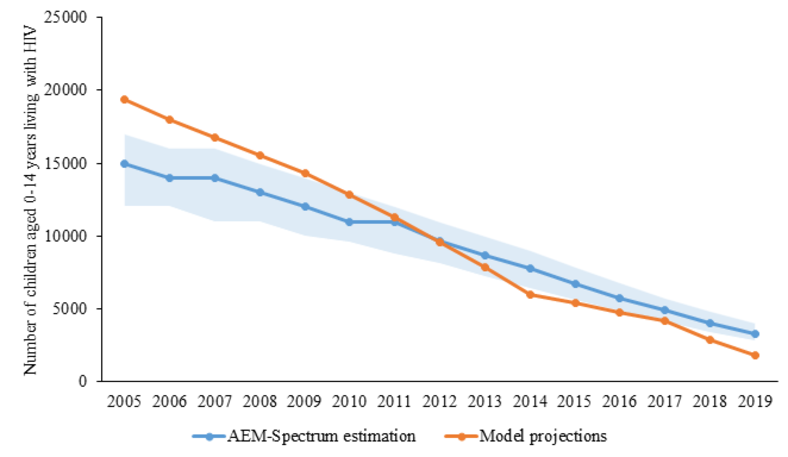

Supplement: S6 Fig — (TIF) [file pone.0276330.s013.tif]

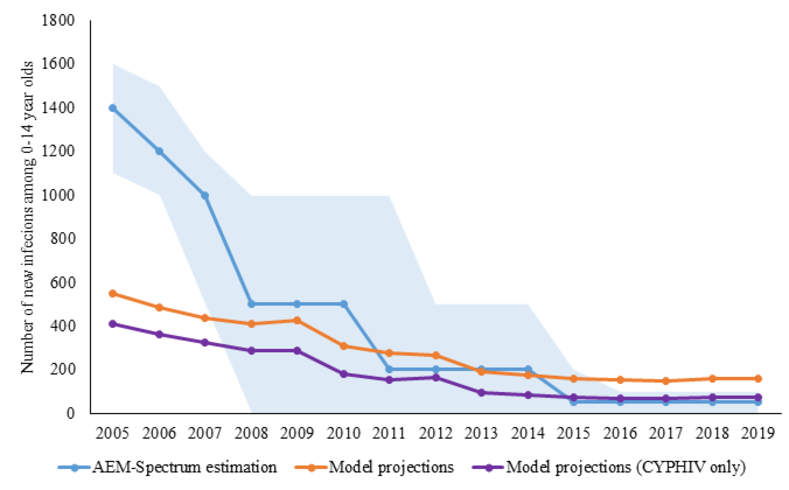

Supplement: S7 Fig — (TIF) [file pone.0276330.s014.tif]

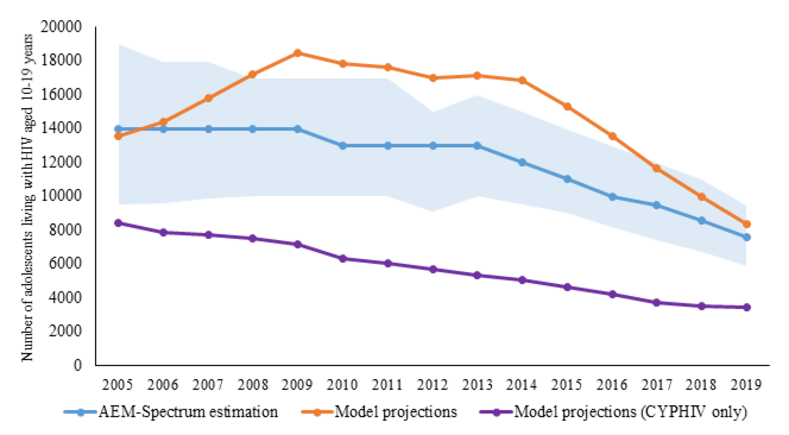

Supplement: S8 Fig — (TIF) [file pone.0276330.s015.tif]

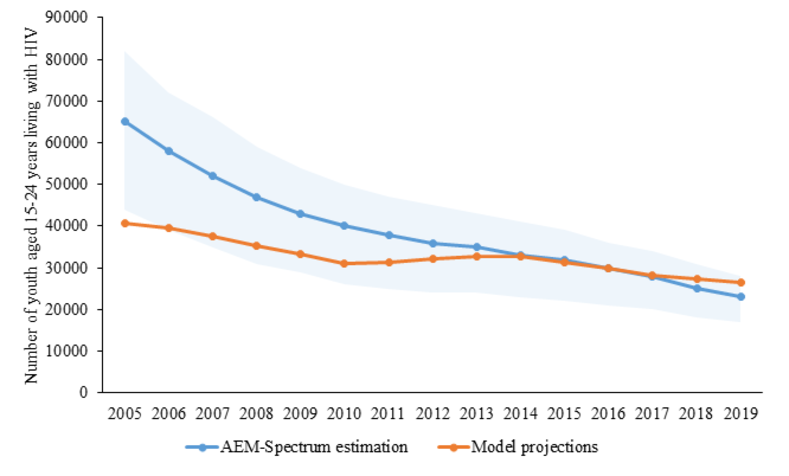

Supplement: S9 Fig — (TIF) [file pone.0276330.s016.tif]

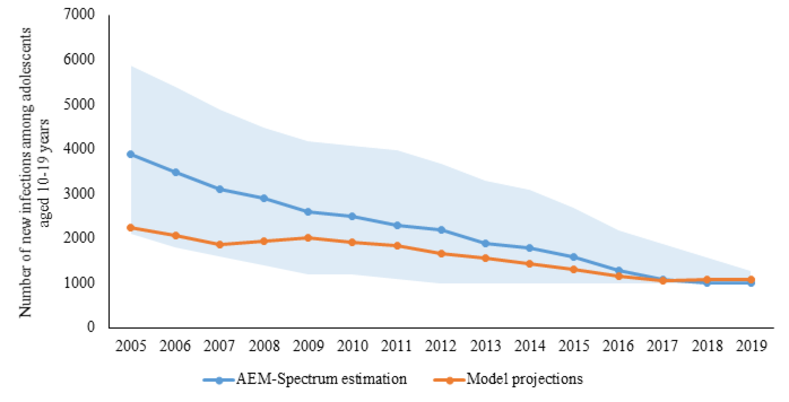

Supplement: S10 Fig — (TIF) [file pone.0276330.s017.tif]

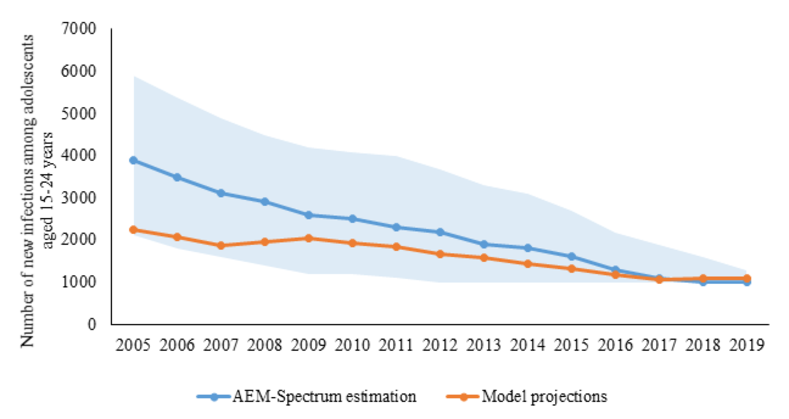

Supplement: S11 Fig — (TIF) [file pone.0276330.s018.tif]

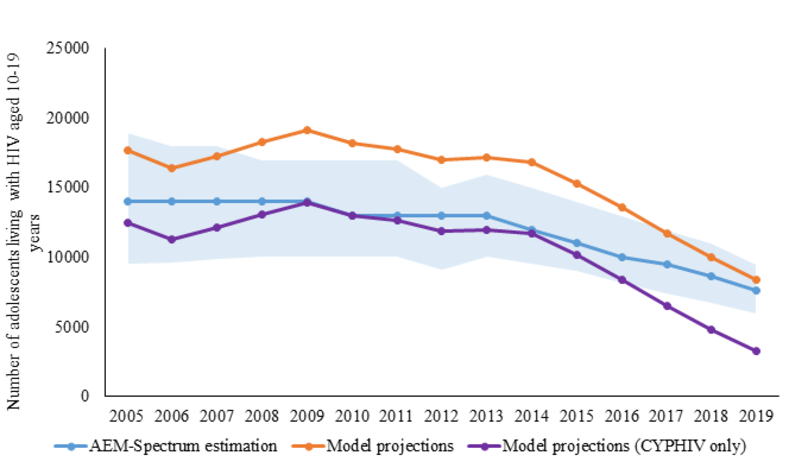

Supplement: S12 Fig — In this sensitivity analysis, the prevalence of HIV in 2005 is doubled compared to the base case. (TIF) [file pone.0276330.s019.tif]

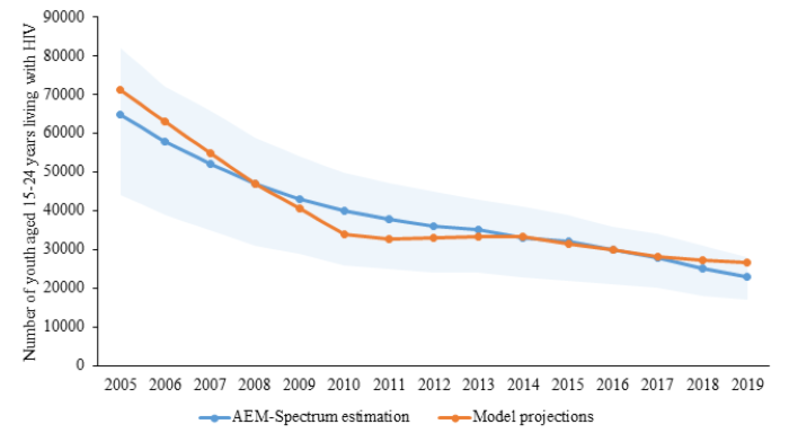

Supplement: S13 Fig — In this sensitivity analysis, the prevalence of HIV in 2005 is doubled compared to the base case. (TIF) [file pone.0276330.s020.tif]

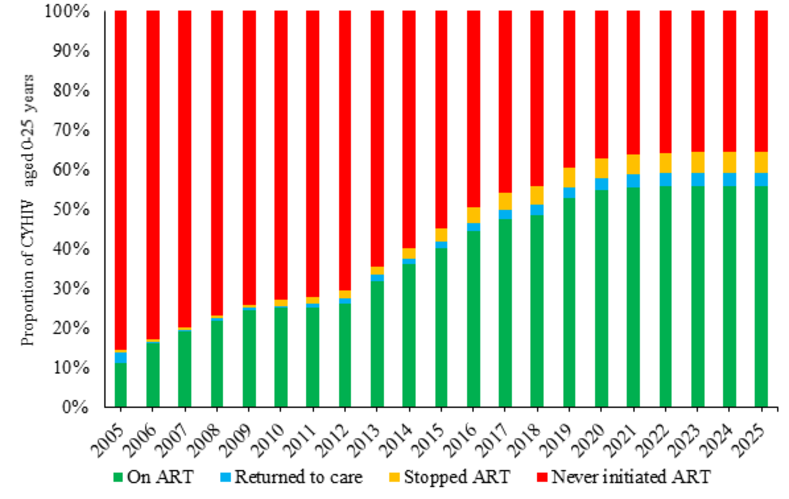

Supplement: S14 Fig — The vertical axis shows the proportion of all CYHIV aged 0–25 years in Thailand who fall into each category of ART coverage (on ART, returned to care, stopped ART, and never initiated ART). The horizontal axis shows each calendar year from 2005 to 2025. ART: antiretroviral therapy, CYHIV: children and youth living with HIV, CYPHIV: children and youth living with perinatally acquired HIV, CYNPHIV: children and youth living with non-perinatally acquired HIV. (TIF) [file pone.0276330.s021.tif]

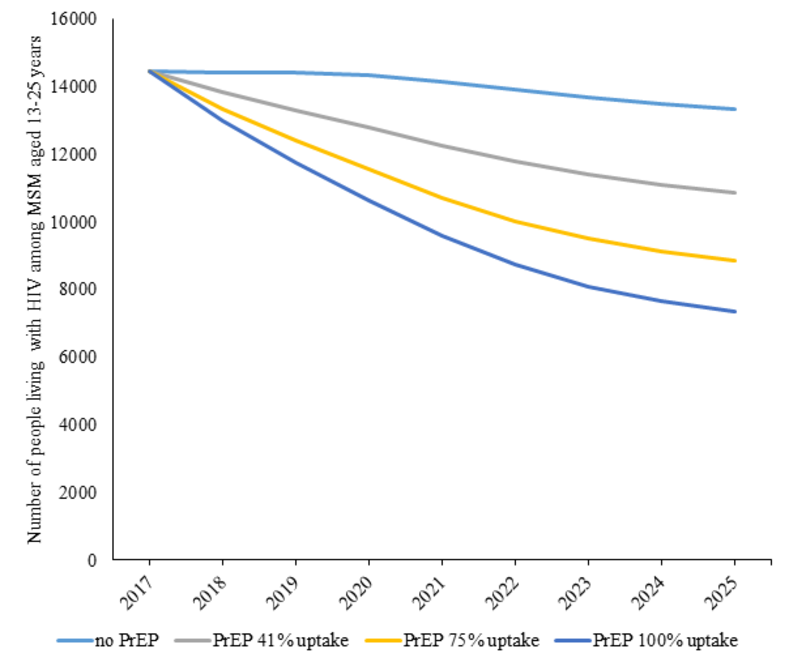

Supplement: S15 Fig — (TIF) [file pone.0276330.s022.tif]
